# Green Chemistry Metrics with Special Reference to Green Analytical Chemistry

**DOI:** 10.3390/molecules200610928

**Published:** 2015-06-12

**Authors:** Marek Tobiszewski, Mariusz Marć, Agnieszka Gałuszka, Jacek Namieśnik

**Affiliations:** 1 Department of Analytical Chemistry, Faculty of Chemistry, Gdańsk University of Technology (GUT), 11/12 G. Narutowicza St., Gdańsk 80-233, Poland; E-Mails: marcel.chem@gmail.com (M.M.); chemanal@pg.gda.pl (J.N.); 2 Geochemistry and the Environment Division, Institute of Chemistry, Jan Kochanowski University, 15G Świętokrzyska St., Kielce 25-406, Poland; E-Mail: aggie@ujk.edu.pl

**Keywords:** green chemistry metrics, green analytical chemistry metrics, environmental impact, E-Factor, atom economy, eco-footprint, Eco-Scale, EATOS

## Abstract

The concept of green chemistry is widely recognized in chemical laboratories. To properly measure an environmental impact of chemical processes, dedicated assessment tools are required. This paper summarizes the current state of knowledge in the field of development of green chemistry and green analytical chemistry metrics. The diverse methods used for evaluation of the greenness of organic synthesis, such as eco-footprint, E-Factor, EATOS, and Eco-Scale are described. Both the well-established and recently developed green analytical chemistry metrics, including NEMI labeling and analytical Eco-scale, are presented. Additionally, this paper focuses on the possibility of the use of multivariate statistics in evaluation of environmental impact of analytical procedures. All the above metrics are compared and discussed in terms of their advantages and disadvantages. The current needs and future perspectives in green chemistry metrics are also discussed.

## 1. Introduction

The concept of green chemistry [1] has become a tool for promoting sustainable development in laboratories and industry. The twelve principles of green chemistry [2] are the basis of guidelines addressed to those who want to follow the green chemistry trend. They provide a framework for actions that can be taken to make chemical products and processes more environmentally benign. These actions are developed by chemists representing different areas of chemistry, for example, organic synthesis, chemical engineering, or analytical chemistry.

Most efforts in making chemical processes greener emphasize the need for using safer, less toxic, and more benign solvents, or the elimination of solvents, and reduction in the use of reagents and auxiliaries. Other actions include lowering energy consumption through the use of milder reaction conditions [3], avoiding derivatization and a preference for substrates based on renewable sources [4]. In order to improve atom economy, highly selective catalytic processes should be performed instead of using additional substrates. These solutions are well-defined and have been intentionally put into practice since 1998 and were known even before their introduction.

One of the challenges in green chemistry is the evaluation of the greenness of chemical processes. It is well known that the processes that cannot be measured cannot be controlled. Control in green chemistry should be understood as a possibility to select the greenest option. The development and application of measurement procedures allows us to compare the greenness of existing solutions with newly developed ones. Different factors characterized by a different level of complexity are currently used in evaluation of environmental impact of chemical processes.

The aim of this article is to critically review different approaches to measuring the environmental impact of chemical processes. The principles, areas of application, advantages and disadvantages of different semi-quantitative, quantitative and comparative assessment procedures will be discussed.

## 2. Green Metrics Commonly Applied throughout the Industry

### 2.1. Eco-Footprint

At the beginning of the 1990s, Rees and Wackernagel introduced and characterized an accounting tool known as Ecological Footprint (EF) or Ecological Footprint Analysis (EFA), which measures the demand on certain resources (ecosystem services) necessary for a defined level of consumption for an industrial process or for a certain building project. Moreover, the EF defines the ability of the ecosystem to absorb the post-consumer waste and to compensate for all the resources used for production of goods and services in a particular area. Global hectare (gha) per person is the unit of the EF measurement. The lower the EF value is, the more environmentally friendly the industrial processes or population’s consumption in the area will be. In the evaluation of the EF six main ecological land-use categories are considered, *i.e*., forest land, fishing ground, arable land, built-up land, grazing land and land used for energy production [5,6,7,8,9,10,11,12,13,14,15]. Currently, aside from general EF, specific EF for individual factors influencing ecosystems are becoming more popular. The examples of specific EF are: Chemical Footprint (Sala and Goralczyky, 2013) [16], Material Footprint (Laakso and Lettenmeier, 2015) [17], Energy Footprint (Vujanovic *et al.*, 2014) [18], Land Footprint (Hsien H. Khoo, 2015) [19], Water Footprint (Mansardo *et al.*, 2014) [20], Carbon Footprint (Rodriguez-Caballero *et al.*, 2015) [21], Nitrogen Footprint (Singh and Bakshil, 2015) [22], Phosphorus Footprint (Wang *et al.*, 2011) [23]. 

**Figure 1:**
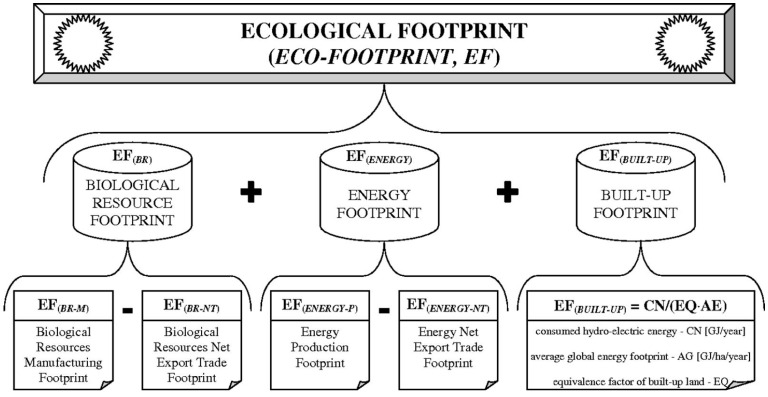
EF evaluation procedure based on Biological Resource Footprint, Energy Footprint and Build-up land Footprint (compiled from Fu *et al.*, 2015) [15].

A novel approach for calculating the general EF parameter has been proposed by Fu *et al.*, 2015 [15]. This method considers three main factors, namely Biological Resource Footprint, Energy Footprint and Build-up land Footprint. A scheme showing the EF evaluation procedure developed by Fu *et al.* is presented in Figure 1. This method was used to assess the technological advancement and economic development in China during a period of 1997–2011.

Another interesting approach to the EF assessment and visualization of individual factors having impact on the environment in a given area has been developed by Leseurre *et al.*, 2014 [7]. This method was applied by the Chimex company for evaluation and visualization of environmental impact of industrial-scale production of an active anti-ageing substance Pro-Xylane™ and a UV-A filter Mexoryl^®^ SX. The tool proposed and described by Leseurre *et al*., focuses on two main areas, *i.e.*, manufacturing footprint and eco-design footprint. Each of these two groups is influenced by five basic indicators assigned on a 0–4 scale. The higher the value, the greater the impact on the environment in a particular area. Factors that are critical for manufacturing footprint values are: (i) water consumption (H_2_O); (ii) raw materials’ geographical origin (iL); (iii) aqueous waste valorization (eFA); (iv) used organic solvents valorization (slOS); and (v) process carbon footprint (eC). The eco-design footprint is mostly affected by: (i) synthetic pathway efficiency (eVS); (ii) raw materials of renewable origin (rMP); (iii) E-Factor (eF); (iv) potential environmental impact of raw materials (ieMP); and (v) potential environmental impact of waste (ieD). Evaluation of all 10 indicators allows drawing a specific type of a radar chart presenting an environmental impact of the industrial process. This method enables the company to estimate how a change in technology of production will affect its environmental impact [7].

The main advantage of the use of ecological footprint as a measure of impact assessment is that it provides a simple, comprehensible and effective tool for evaluation of the environmental impact of production and consumption. Moreover, this metric can be used to evaluate the effect of industrial activity on any scale—from a local environment (on a district or regional scale) to the whole country or even a continent.

### 2.2. E-Factor

Considering the fact that the simplest solutions are the best, Sheldon has developed a simple and fast metric for evaluation of environmental impact of industrial processes, referred to as E-Factor (environmental factor). According to its definition, the E-Factor is calculated as a total weight of all waste generated in technological or industrial process (in kilograms) per kilogram of a product. The closer to zero the value of E-Factor (E-Factor ~0) is, the less waste generated and more sustainable and greener the process will be. However, it should be realized that, depending on its potential application, the E-Factor can be calculated including or excluding water used in the process [24,25,26,27,28]. Table 1 presents the published values of E-Factor calculated for selected chemical industry sectors. This parameter can also be used for evaluation of environmental impact of a specific industrial process, for example production of a certain electronic device [29].

**Table 1:** The numerical values of E-Factors in different chemical industry sectors [25].

Industry Sector	Product Tonnage	E-Factor (kg Waste/kg Product)
Oil refining	10^6^–10^8^	<0.1
Bulk chemicals	10^4^–10^6^	<1.0 to 5.0
Fine chemicals industry	10^2^–10^4^	5.0 to > 50
Pharmaceutical industry	10–10^3^	25 to > 100 (25 to >200 *)

* data proposed by Dunn associated with a so-called ‘chiral era’ of pharmaceuticals [29].

Higher E-Factor values reported for pharmaceutical industry compared with these values for other sectors of chemical industry result from a necessity to obtain a very high-purity product in the multi-stage reactions during which many by-products (waste) are generated. Additionally, production of pharmaceuticals requires the use of high-purity reagents [30].

A major limitation of the E-Factor as a metric of environmental impact of technologic process is that it neither considers the hazards nor the environmental risk of the produced waste. The two examples of successful application of the E-Factor to evaluation of the greenness of technologic process in pharmaceutical industry are: synthesis of sildenafil citrate (Viagra™) [30] and synthesis of antidepressant sertraline hydrochloride (Zoloft^®^) [27,31]. In the case of sildenafil citrate synthesis, introduction of toluene and ethyl acetate recovery as well as total elimination of highly volatile solvents (e.g., acetone, diethyl ether) from the synthetic pathway, resulted in lowering of the E-Factor value from 105 (in the time of drug discovery) to 7 in the production stage. The pharmaceutical company that produces Viagra™ has established a future target of lowering the E-Factor value to 4, which would be possible to achieve through elimination of titanium chloride, toluene and hexane [30]. By re-designing the chemical process the manufacturers of sertraline hydrochloride (Zoloft^®^) achieved the E-Factor value of 8 [27,31].

The E-factor is a versatile metric that can be used in various chemical industry sectors, such as inorganic synthesis. For example, Demirci and Miele used the E-Factor to evaluate the greenness of 11 methods of hydrogen production. The calculated E-Factor values were in the range of 5.5 (for steam reformation from natural gas—methane) to 16.5 (for thermolysis/gasification process in which coal was the main hydrogen source) [32].

In some cases, more than one green metric is used for evaluation of environmental impact of an industrial process. E-Factor and the Product Mass Intensity (PMI) can be given as an example of such multi-metrics approach. The PMI has found its widest application in pharmaceutical industry. A relation between E-Factor and PMI can be described by the following formula: E-Factor = PMI − 1. It is easier to calculate the PMI than E-Factor because it only requires the knowledge about inputs into a reaction [33,34].

The E-Factor value is necessary for calculation of another popular green chemistry metric, the Environmental Quotient (EQ). It is a product of E-Factor and a Q value. The Q value is defined as Environmental Hazard Quotient. It is related to ecotoxicity of waste generated during an industrial process or organic synthesis. For example, the Q value for sodium chloride is 1 whereas this value for heavy metals and their salts is in the range of 100–1000 [35].

## 3. Green Metrics Applied to Organic Synthesis

The principles of green chemistry have the highest impact on organic synthesis performed in laboratory and in industry. An ideal synthesis should have as little steps as possible, be characterized by a high selectivity and be based on using easily available and inexpensive substrates [36]. For implementation of the green chemistry principles it is important to modify and improve known synthesis pathways in order to make these syntheses more environmentally friendly and sustainable. Designing synthesis of new compounds requires the use of green substrates, green reagents and green reaction conditions. In general, green chemistry in organic synthesis aims at: (i) reduction of the volume of waste and by-products that are generated during each step of synthesis; (ii) elimination of toxic reagents and solvents; (iii) replacement of the solvents by their green alternatives such as water, supercritical fluids, ionic liquids *etc.*; (iv) the use of catalysts, such as photocatalysts [37,38]. An important issue in green organic synthesis is reduction of energy use, which is possible through replacing traditional heating of the reaction mixture with alternative energy sources such as microwave irradiation, ultrasonication or irradiation under specific light wavelengths. This approach often leads to additional benefits such as an improvement of the reaction efficiency, an increase of reaction rate, and a reduction of the waste volume.

Since the beginning of the 1990s, a new trend in green organic synthesis has emerged. This concerned developing metrics for evaluation of environmental impact of the whole process or its individual steps. The main purpose of these metrics is to obtain clear, simple and fast information about the greenness of organic synthesis or its specific steps. They also enable to predict how a certain change in a synthesis path, such as elimination or replacement of a solvent, would influence its environmental impact [26]. Table 2 summarizes several metrics used for evaluation of the greenness of organic syntheses with their short characteristics.

**Table 2:** Examples of green chemistry metrics applied to organic synthesis.

Parameter	Formula	Short Characteristics	Comments	Ref.
***Carbon Efficiency (CE)***	$\begin{array}{l} A+B\to D\\ \%CE=\left(\frac{\text{amount of carbon in prouct D}}{\text{total carbon presented in reactants}}\right)\times 100 \end{array}$	It is used to estimate the percentage of carbon in the reagents used in organic synthesis that remain in the final desired product	This parameter is dedicated to evaluation of the greenness of organic synthesis based solely on carbon accounting	[26,39]
	$\begin{array}{l} A+B\to D+F\\ \%CE=\left(\frac{\text{no. of moles of D}\times \text{no. of carbon in D}\times \text{100}}{\text{moles in A}\times \text{carbon in A}+\text{moles in B}\times \text{carbon in B}}\right) \end{array}$			
***Effective Mass Yield (EMY)***	$\%EMY=\frac{\text{mass of final product}}{\text{mass of hazardous and toxic reagents}}$	This parameter quantifies a percentage of the final product in all reagents and materials used in organic synthesis	Reagents having low or very low environmental impact (e.g., sodium chloride or acetic acid) are excluded from calculation of EMY	[40,41]
***Mass Intensity (MI)***	$\begin{array}{l} \left[\frac{kg}{kg}\right]MI=\frac{\text{total mass used in a process}}{\text{mass of final product}}\\ \text{Mass productivity}=\frac{\text{1}}{MI}\times \text{100} \end{array}$	The MI takes into account reaction efficiency, stoichiometry, amount of solvents, all reagents and auxiliary substances used in synthesis.	This parameter has a value of 1 for an ideal synthesis, in which the total mass of input is equal to the mass of product	[26,39]
***Reaction Mass Efficiency (RME)***	$RME=\frac{1}{1+E_{m}}$ *where E_m_ is a value of E-factor based on mass*	The RME factor is inversely related to the overall E-factor described by Sheldon. The RME offers a better and easy way of identification of the best or the worst reactions that have influence on whole industrial process or synthesis.	This parameter was described very precisely by Andraos and Sayed (2007). The final version of RME equation depends on conditions of reaction or process (recovery of reaction solvents or post-reaction materials). This parameter is most effective in efforts to reduce waste at the intrinsic and global level	[39,42,43,44,45,46]
	$RME=(\varepsilon )AE\frac{1}{SF}\left[\frac{1}{1+\frac{\varepsilon AE(c+s+w)}{SFm_{cp}}}\right]$ *where: —reaction yield; AE—atom economy; SF—stoichiometric factor; c—the mass of reaction catalyst; s—the mass of reaction solvent; w—the masses of all other post-reaction materials; m_cp_—the mass of the collected target product*			
***Atom Utilization (AU)***	$\%AU=\frac{\text{mass of the final product}}{\text{total mass of all the substances produced}}$	This parameter defines percentage ratio of the mass of final product to the mass of all products (final product and byproducts) obtained in synthesis. The solvents are excluded from calculations	It provides fast and simple evaluation of the greenness of a process or individual reaction in terms of produced waste. Nowadays it is seldom used	[39,47]
***Solvent and catalyst environmental impact parameter (f)***	$\text{f}=\left(\frac{\sum \begin{array}{l} \text{mass of reaction and postreaction solvents}\\ \text{and materials}+\text{mass of catalysts used} \end{array}}{\text{mass of final product}}\right)$	Evaluation of this parameter takes into account actual masses of materials used in the process	This parameter has a value of 0 only if all materials (solvents, catalysts *etc.*) used in the process or in individual step of synthesis are recycled, recovered or eliminated. In every other case, f > 0	[42,43,44,48]
***Stoichiometric Factor (SF)***	$SF=\text{1}+\left(\frac{\left(AE\right)\sum \text{mass of excees reagents/chemicals}}{\text{expected product mass at 100\% yield}}\right)$	This parameter is calculated in case of syntheses in which one or more reagents are used in excessive amount	The SF has a value of 1 for stoichiometric reactions. If the reaction is nonstoichiometric the SF > 1	

One of the most important tool which can be considered as a fundamental green chemistry metric that forms the basis for all of the other metrics is Atom Economy. This measure was introduced in 1991 by Trost and it is the simplest, fundamental and the most popular parameter used in drug synthesis. Atom Economy calculation (Equation (1)) estimates the amount of reagents (substrates, solvents, catalysts) that will be incorporated into the final desired product [26,49]:

(1) $$\begin{array}{l} A+B\to C\\ \%AE=\left(\frac{\text{molecular weight of C}\times \text{100}}{\text{molecular weight of A}+\text{molecular weight of B}}\right) \end{array}$$

One of the tools for measuring the greenness of synthesis is reaction mass efficiency (RME). It is a comprehensive tool in terms of mass balance of a chemical process [42]. Reaction yield, atom economy and stoichiometric factor taking into account the excess of reagents, are included in calculation of RME. Amounts of auxiliary compounds, solvents, catalysts, as well as a recovery of these compounds after reaction are also considered. The calculation can easily be carried out by using the Excel spreadsheet. The results of analysis can be visualized to facilitate the actions to improve the greenness of chemical process [43]. The RME value is usually significantly influenced by the treatment of solvents and auxiliaries that are used during the reaction [48]. Another drawback is a difficulty in proper identification of all by-products, which is a prerequisite of RME analysis [44]. For multi-stage reactions it would be advisable to apply a tree analysis for making a comparison easier or even for ranking of synthesis plans [50]. Eissen and Metzger proposed another tool referred to as Environmental Assessment Tool for Organic Syntheses (EATOS) for an assessment of the greenness of a laboratory-scale organic synthesis. It is a software capable of comparison of different methods of the same product synthesis in terms of their environmental impact, the use of resources, identification of the least environmentally friendly steps *etc*. The EATOS tool provides a comprehensive analysis of the greenness of all the chemicals used in synthesis, *i.e*., solvents, sewage/water, impurities, catalysis, auxiliary materials, byproducts, coupled products and substrates [51]. Information needed for performing EATOS analysis can easily be found in the substance specification sheets. More details about this tool and examples of its use have been presented by many authors [52,53,54,55].

The EATOS software has been used for comparison of the greenness and environmental impact of six different methods of synthesis (including thermal and photochemical processes) of a mixture of four diastereoisomers: *cis*- and *trans*-4-methyl-2-(2-methylprop-1-en-1-yl)tetrahydro-2*H*-pyran) commonly known under the name ‘rose oxide’. The lowest EATOS value (in PEI kg^−1^, where PEI is Potential Environmental Impact per kg of product), 29.24 was obtained for Dragoco protocol. The highest EATOS value of 467.81 PEI kg^−1^ was found for synthesis conducted with oxidants [53]. Similarly, Protti *et al*. evaluated the environmental impact of photochemical and thermal synthesis of methyl cyclohexylpropanoate, alkynylbenzoxazole and β-hydroxyl ketone using EATOS [56]. It is interesting to note that this software has also found its application in the field of inorganic synthesis. For example, Pini *et al.* used EATOS to evaluate the greenness of TiO_2_ nanoparticle synthesis [54].

In 2006 Van Aken *et al*., introduced the EcoScale, which is a semiquantitative tool to evaluate the quality of the organic preparation on a laboratory scale. This approach is focused on several parameters that characterize organic synthesis, such as yield, cost, safety, conditions and ease of workup/purification. The highest rank in the EcoScale (100 points) refers to ‘ideal’ reaction that has a 100% yield, uses inexpensive reagents and is conducted at room temperature, is safe for both the operator and for the environment. If the parameters of a real synthesis differ from ‘the ideal value’, the penalty points are assigned, lowering the total score by certain values that have been proposed by Van Aken *et al*. [57]. The EcoScale value allows to select different preparations according not only to their greenness, but also to their costs. The main disadvantage of the EcoScale evaluation is that it does not provide information about the type of hazards, it only gives a score.

## 4. Green Analytical Chemistry

Green analytical chemistry has its own achievements in measuring an environmental impact of analytical processes. The analytical process refers to determination of certain substances, it differs from the industrial processes mostly by its scale. In contrary to industrial emissions, analytical processes cause a dispersed pollution. The emissions from analytical laboratories are on one hand low, but on the other hand, are more dispersed than industrial emissions, which makes them more difficult to control.

The main goal of an industrial chemical process is to obtain a certain product. All the inputs to the process, which do not form the final product are considered as waste. Analytical processes lead to obtaining a very specific product, namely results of analyses. Therefore, all the material and energetic inputs to the analytical process contribute to generation of waste. Analytical waste cannot be fully eliminated, but its volume can be reduced. The environmental impact of analytical methods/procedures and their alternatives has to be appropriately measured.

**Figure 2:**
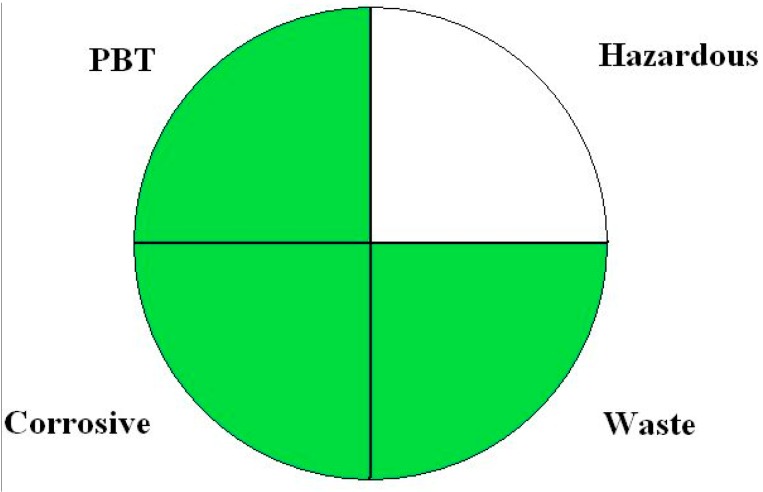
The example of NEMI pictogram. The field is green if the requirements of criterion are fulfilled.

One of the oldest tools to assess the greenness of analytical procedures is NEMI labeling [58]. The NEMI label is a circle consisting of four fields. Each field reflects different aspect of the described analytical methodology and the field is filled green if certain requirements are met (the reader is kindly referred to Figure 2). The first requirement is that none of the chemicals is present on the persistent, bioaccumulative and toxic chemicals list. The second of the requirements is that none of the chemicals applied in the procedure is listed on D, F, P or U hazardous wastes lists. The third requirement is that the pH of the sample is within 2–12 range to avoid a highly corrosive environment during the whole analytical process. The fourth and last requirement is that during the procedure less than 50 g of waste is produced. 

The main advantage of the NEMI as a greenness assessment tool is that it is easy to read by potential procedure users. One glance at the NEMI symbol is enough to have a general information about an environmental impact of a procedure. The two main disadvantages of NEMI labeling are that the obtained information is rather general and that filling the NEMI symbol is time consuming. NEMI symbol shows that each threat is below or above a certain value. Therefore it cannot be regarded as being semi-quantitative. The second disadvantage is connected with a tedious preparation of a symbol, especially if many, non-typical chemicals are used in the procedure. Every chemical has to be checked if it is present on at least one of the few lists.

An improvement to the NEMI pictogram was proposed by de la Guardia and Armenta [59]. These authors suggested that each of the fields should be colored using a three degree scale—red for non-environmentally friendly analysis, yellow for moderate and green for environmentally benign analysis. This modification makes the NEMI procedure assessment more quantitative.

The analytical Eco-Scale is another approach to the assessment of environmental impact of analytical methods [60]. The result of analytical Eco-Scale analysis is the score that is calculated by subtracting penalty points from the basis of 100 points. The penalty points are assigned for high amounts and high hazards connected with utilization of chemicals, high energy consumption, occupational hazards and generation of wastes. The summary of the procedural penalties is presented in Table 3. A final result of analytical Eco-Scale assessment is a number differing from 100 (“ideal green analysis”) by a number of penalty points. The higher the value (closer to 100) is, the greener analysis will be.

**Table 3:** Penalty points applied for the calculation of final analytical Eco-Scale score.

		Sub-Total Penalty Points	Total Penalty Points
Reagents			
Amount	<10 mL (<10 g)	1	Amount penalty points × hazard penalty points
	10–100 mL (10–100 g)	2	
	>100 mL (>100 g)	3	
Hazard	None	0	
	Less severe hazard	1	
	More severe hazard	2	
Instruments			
Energy	<0.1 kWh per sample		0
	<1.5 kWh per sample		1
	>1.5 kWh per sample		2
Occupational hazard	Hermetization of analytical process		0
	Emission of vapors to the atmosphere		3
Waste	None		0
	<1 mL (<1 g)		1
	1–10 mL (1–10 g)		3
	>10 mL (>10 g)		5
	Recycling		0
	Degradation		1
	Passivation		2
	No treatment		3

The penalty points for each reagent are calculated by multiplying number of GHS hazard pictograms by degree of hazard (‘warning’ multiplication by 1 and ‘danger’ multiplication by 2). Because the GHS hazard pictograms are placed on the reagent containers, the hazard related to utilization of chemicals is easy to calculate. The examples of penalty points calculations for solvent and reagent use are presented in Table 4.

**Table 4:** The penalty points for selected analytical solvents and reagents.

Solvents/Reagents	Pictograms	Signal	Penalty Points
dichloromethane	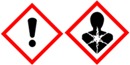	warning	2
hexane	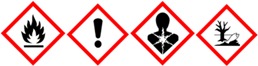	danger	8
diethyl ether	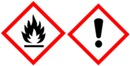	danger	4
methanol	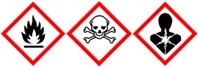	danger	6
ethyl acetate	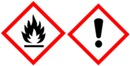	danger	4
MTBE	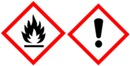	danger	4
acetone	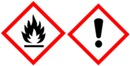	danger	4
benzene	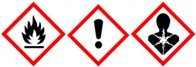	danger	6
isooctane	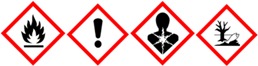	danger	8
acetonitrile	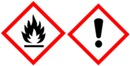	danger	4
isopropanol	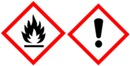	danger	4
toluene	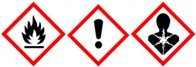	danger	6
chloroform	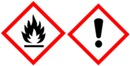	danger	2
elemental mercury	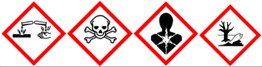	danger	8

The advantages of the analytical Eco-Scale are: ease of its score calculation, ease of comparison of analytical procedures and including different aspects of environmental impact in its assessment procedure. On the other hand, no information about the structure of the hazards is obtained. The final result of Eco-Scale calculation does not inform about the cause of environmental impact of analytical procedure such as the use of solvents, other reagents, occupational hazard or generation of wastes. Compared to NEMI labeling, analytical Eco-Scale provides information about environmental impact of analytical procedures in a more quantitative way. The amounts of reagents and wastes are considered, not only the certain threat occurrence or its lack. 

A tool dedicated to identification of hazards related to the use of liquid chromatographic mobile phases is the high performance liquid chromatography—environmental assessment tool (HPLC-EAT) [61]. Evaluation of HPLC-EAT includes safety, health and environmental factors (SHE approach) and a weight of every chemical that is used in the liquid chromatographic run and during sample preparation. An assessment algorithm involves diverse parameters and hazards of solvents used as mobile phases, which makes the procedure comprehensive. The disadvantage is that a result is a single number that gives the general view, but does not provide any information about a character of threat. On the other hand, the software is easily available and easy to use.

Metrological parameters such as a time of analysis, are usually considered for comparison of different analytical procedures. Environmental aspects represent an added value when comparing analytical methods. Ruiz-de-Cenzano *et al*., [62] compared two analytical procedures of mercury determination in mushroom samples. The comparison of direct thermal degradation with atomic absorption spectroscopy and microwave-assisted mineralization with cold-vapor atomic fluorescence spectroscopy showed that the two procedures did not differ much in terms of metrology, but they showed significant differences in environmental impact. Analysis of their greenness with Eco-Scale procedure revealed that the score for the former procedure is 92, while for the latter one is only 59. It is not unusual that for a given analyte determination in certain sample matrix there are dozens of analytical procedures developed and available for the analyst. In this case a pair-wise comparison can be very difficult and application of extra tools may be required. There is a set of techniques, called multivariate statistics, which allows us to group objects and variables that describe the objects according to their similarity [63]. For example, the self-organizing maps [64], representing one of the most beneficial multivariate statistics tools [65] were used for grouping analytical procedures for determination of benzene (26 procedures) and phenol (21 procedures) in water samples [66]. These datasets were characterized by: limit of detection, sample volume, injection volume, number of analytes determined in a single run, amount of organic solvents used, amount of solid waste generated and NEMI as well as Eco-Scale scores as the variables. The analysis has shown that the main discriminators are those related to environmental impact of the methodologies. Another finding of that study is that the results of methodologies assessment with NEMI and Eco-Scale tools are well correlated. The self-organizing maps procedure was also applied to investigation of the dataset consisting of 43 procedures for the aldrin determination in water samples [67]. The findings were similar and they showed that the factors responsible for a negative environmental impact are those that make the statistical difference among the procedures. The other important conclusion was that the standard procedures were categorized as causing serious environmental problems and that the less controversial substitutes were available. The main advantage of multivariate statistics as an assessment tool is a possibility to apply complex description of a dataset by selecting several important variables relevant to the problem. Environmental variables and other parameters can be included in the assessment, making grouping of the procedures more comprehensive. An advanced multivariate statistics user may find many interesting relations between variables and objects included in the dataset. The main disadvantage is a tedious preparation of the dataset and an advanced assessment procedure. Another drawback is that the analytical procedures are only grouped according to their similarity, so exhaustive interpretation of the results is still required. In contrary to NEMI labels and Eco-Scale, it cannot be considered as a routine assessment procedure.

Multivariate statistics only allow for analytical procedures to be grouped according to their similarity, while multicriteria decision analysis (MCDA) tools allow ranking of the procedures. The assessment procedure with MCDA involves finding the possible alternative solutions to a certain problem, setting the criteria, assigning them weights (usually this step needs a survey among experts) and running the algorithm. As a result, the ranking of the solutions is formed, so the procedures are listed according to the analyst preferences. What is more, apart from ranking, the numerical values are given for each alternative analytical procedure, so an additional information can be obtained in the form of “similarity to ideal solution”. There are several basic techniques developed—TOPSIS [68], AHP [69], ELECTRE [70] and PROMETHEE [71], but so far only the last mentioned has been applied to an assessment of analytical procedures [72]. One of the crucial steps in MCDA techniques is weighting the criteria, so it depends on expert’s preferences whether the environmental criteria are considered as important or not. The results of procedures ranking may vary from expert (analyst) to expert (analyst). If MCDA techniques are seriously considered as a routine greenness assessment tool, some improvements in terms of unifying the criteria and assigning them weights should be elaborated. The main advantage of PROMETHEE as a green analytical chemistry metrics tool is the ability to obtain the full ranking of the procedures. The disadvantages are similar to those of multivariate statistics. The dataset preparation is rather difficult and requires some analytical knowledge and experience.

## 5. Education in Green Chemistry

Green chemistry is not the branch of science itself but it is an added value to organic chemistry, technology or analytical chemistry [73]. Nowadays it is more often included in university courses and programs. Students should know the greenness assessment tools described to properly assess the environmental impact of chemical processes [74]. Green chemistry teaching in student laboratories means incorporation of environmental factors into optimization of chemical processes. Students should be aware how to describe the environmental impact of the reactions and how try to find greener alternatives [75,76,77]. Even the complex ideas, like LCA, are introduced to teaching students on how to assess the greenness of syntheses [78] and a novel approach to this field has recently been proposed [79].

There is also a special tool dedicated to green chemistry education. The “Green Star” tool is used to describe the greenness of organic synthesis in student laboratories. With this approach, the students can get information on how to improve the greenness of the existing reactions. The assessment tool is used to measure the greenness of both the initial and improved reaction. The basic concept of the Green Star is presentation of the twelve green chemistry principles, each as an arm of the star, with the arm’s length corresponding to the fulfillment of the principle [80]. To obtain the length of the arms, semi-quantitative scores are calculated for each of the twelve principles (sometimes less than twelve if the criteria are not applicable). Green Star allows easy identification of the weak points of the synthesis to compare two or more procedures with each other. The drawback is that some information, such as degradability or hazards, is not easily available. For further information about teaching green chemistry, including green chemistry metrics, please refer to the excellent review by Andraos and Dicks [81].

## 6. Conclusions

Together with the development of green chemistry, new green chemistry metrics are and should be introduced. Two future trends in the green chemistry metrics development can be predicted. The first one is an introduction of assessment methods that are very simple, easy to apply and easy to interpret. The second one is establishing the greenness models, in which large input datasets are used. At the same time, it is expected that the new software and other tools that make calculation of greenness parameters easier, will be developed. There is a need for introduction of new tools to evaluate the greenness of different processes and on different scales. There is also a need for popularization of the existing green chemistry metrics. Thus, groups of professionals such as organic chemists, chemical process engineers, analytical chemists and educators should contribute to this field in order to establish universally applied greenness assessment procedures.
